# The expression of Ki-67 and Glypican -3 in hepatocellular carcinoma was evaluated by comparing DWI and ^18^F-FDG PET/CT

**DOI:** 10.3389/fonc.2023.1026245

**Published:** 2023-10-18

**Authors:** Xuedong Wang, Lei Li, Linjie Wang, Min Chen

**Affiliations:** ^1^ Department of Radiology, Zhuhai People’s Hospital (Zhuhai Hospital Affiliated Jinan University), Zhuhai, China; ^2^ Department of Nuclear Medicine, Zhuhai People’s Hospital (Zhuhai Hospital Affiliated Jinan University), Zhuhai, China; ^3^ Department of Pathology, Zhuhai People’s Hospital (Zhuhai Hospital Affiliated Jinan University), Zhuhai, China

**Keywords:** Ki-67, Glypican-3, MRI, 18 F-FDG PET/CT, hepatocellular carcinoma (RGB(46, 48, 51))

## Abstract

**Objective:**

The value of DWI and ^18^F-FDG PET/CT in evaluating the expression of Ki-67 and GPC-3 in HCC was compared.

**Materials and methods:**

Ninety-four patients with primary HCC confirmed by pathology were retrospectively divided into high- and low-Ki-67-expression groups and positive- and negative- GPC-3 groups. The ADC and SUVmax values of the lesions in both groups were measured. ROC curves were used to evaluate the identification efficiency of parameters with significant differences for each group of lesions, and AUCwas calculated. The combined ADC and SUVmax values were analyzed by binary logistic regression. The Delong test was used to compare the AUC values of the combined and single parameters. Pearson (in line with normal distribution) or Spearman (in line with abnormal distribution) correlation analysis was used to analyze the correlation.

**Results:**

The ADC value of the high-Ki-67-expression group was lower than that of the low-Ki-67-expression group (*P*<0.05), and the SUVmax value of the high-expression group was higher than that of the low-expression group (*P*<0.05). The ADC value of the positive-GPC-3 group was lower than that of the negative group (*P*<0.0.tive group (*P*<0.05). The combined ADC and SUVmax values in the GPC-3 group were better than those of a single parameter (*P*<0.05). There was a strong negative correlation between the SUVmax value and ADC value in the Ki-67 group (R=-0.578, *P*<0.001) and a weak negative correlation between the SUVmax value and ADC value in the GPC-3 group (R=-0.279, *P*=0.006). The SUVmax value was strongly positively correlated with the Ki-67 expression index (R=0.733, *P*<0.001), while the ADC value was strongly negatively correlated with the Ki-67 expression index (R=-0.687, *P*<0.001).

**Conclusion:**

DWI and ^18^F-FDG PET/CT can be used to evaluate the expression of Ki-67 and GPC-3 in HCC, and there is a certain correlation between the ADC value and SUVmax. Combined DWI and ^18^F-FDG PET/CT is superior to a single technique in evaluating the expression of GPC-3 in HCC patients. However, the combined model did not benefit the Ki-67 group.

## 1 Introduction

Hepatocellular carcinoma (HCC) is the most common primary liver cancer and the fourth leading cause of cancer-related death worldwide (1, 2). Despite the tremendous efforts of medical workers around the world in the treatment of HCC, the prognosis is unsatisfactory (3). The antigen Ki-67, also known as the monoclonal antibody Ki-67, is a cell proliferation-related protein encoded by the MKI67 gene. Ki-67 is expressed in the G1, S, G2 and mitotic phases of the cell cycle and can be used as an indicator of cell proliferation evaluation by immunohistochemical staining. Glypican (GPC)-3 is a heparan sulfate glycoprotein on the surface of the cell membrane. GPC-3 adhesion to the cell membrane is closely related to the occurrence, development and prognosis of HCC (4, 5). A previous study (6) demonstrated that GPC-3 can be used as a more specific and reliable biomarker for the diagnosis of HCC than α-fetoprotein(AFP). GPC-3 is overexpressed in liver cancer tissues, and only a small amount is expressed in healthy liver tissues. Therefore, GPC-3 can be used as an important immunotherapy target for liver cancer (7). In recent years, with the improvement of medical detection technology, an increasing number of scholars believe that the molecular typing of tumors is closely related to the formulation of treatment plans and prognosis. Therefore, clinical detection of the expression of Ki-67 and GPC-3 will be helpful for the treatment and prognosis evaluation of HCC patients. However, the expression levels of Ki-67 and GPC-3 are mostly obtained by immunohistochemical analysis after surgery. If the expression of Ki-67 and GPC-3 could be evaluated before surgery, the indirect method to determine the proliferation of HCC cells would be of positive significance for guiding the rational formulation of clinical treatment plans and predicting the prognosis of patients. As the most commonly used and mature functional imaging technology in Magnetic resonance imaging (MRI), Diffusion weighted imaging (DWI) has been applied in multiple systems to identify benign and malignant tumors, evaluate tumor grading, differentiation and chemotherapy efficacy, and evaluate tumor immunohistochemical expression. ^18^F-Fluoro-2-deoxyglucose(^18^F-FDG) Positron emission tomography/Computed tomography (PET/CT), as the mainstream technology in nuclear medicine, integrates the functional information of PET with the anatomical information of CT. As a tracer, ^18^F-FDG can reflect glucose uptake, while tumor cells are often metabolically active, and their glucose uptake increases, showing a high metabolic focus. As a semiquantitative unit of PET/CT, the SUVmax value can reflect the degree of malignancy and metabolism of tumors. ^18^F-FDG PET/CT shows certain advantages in the early diagnosis, differential diagnosis, staging, grading, differentiation and evaluation of the immunohistochemical expression of tumors. Patients with liver cancer often undergo MRI and ^18^F-FDG PET/CT examination before surgery, although they have different purposes. However, whether the two imaging techniques can complement each other in the preoperative evaluation of immunohistochemical expression of HCC remains to be determined. Therefore, this paper explored the comparative study of DWI and ^18^F-FDG PET/CT in the evaluation of Ki-67 and GPC-3 in HCC.

## 2 Materials and methods

### 2.1 Patients

Patients who received medical treatment at Zhuhai People’s Hospital (Zhuhai Hospital affiliated with Jinan University) from February 2017 to December 2021 and met the following inclusion criteria were retrospectively collected: (1) Patients with primary HCC were confirmed by surgical pathology, clinicopathological data were complete and detailed, and postoperative immunohistochemical indexes included Ki-67 and GPC-3. (2) There were no contraindications for the use of contrast agents, and preoperative DWI and ^18^F-FDG PET/CT were performed simultaneously. (3) The patients had a good respiratory condition and good image quality. The exclusion criteria were as follows: (1) preoperative interventional therapy or chemotherapy and (2) recent history of blood transfusion or hemochromatosis. A total of 94 patients were enrolled, including 85 males and 9 females aged from 31 to 72 years, with an average age of 53.4 ± 7.9 years.

### 2.2 MRI examination

Conventional MRI and DWI scans were performed using a GE Signa HDxt 3.0T MR. The scanning sequence and parameters were as follows: ① axial T1WI sequence:Epetition time(TR)/Echo time(TE) 5.4/2.5 ms, matrix 256×200, Field of view(FOV) 380 mm×340 mm, layer thickness 5 mm, layer interval 1.0 mm, scanning time 1 ‘16 “; ② axial T2WI sequence: TR/TE 5.4/2.5 ms, matrix 256×200, FOV 380 mm×340 mm, layer thickness 5 mm, layer interval 1.0 mm, scanning time 1’ 16” TR/TE 13043/71 MS, matrix 320×320, FOV 380 mm×380 mm, layer thickness 5 mm, layer interval 1.0 mm, scanning time 2 ‘37 “; ③ axial DWI sequence: TR/TE 5455/82 ms, matrix 128×128, b= 0,1500 s/mm2, FOV 380 mm×320 mm (axial position), layer thickness 5 mm, layer interval 1.0 mm, scanning time 2 ‘22 “; and (4) axial and sagittal liver acquisition with volume acceleration (LAVA) dynamic enhanced scan: TR/TE 5.4/1.9 ms, reverse angle 15°, matrix 256×200, FOV 380 mm×350 mm, layer thickness 4.0 mm, layer interval 2.0 mm, acquisition time 3 ‘16 “. Magnevist (Bayer Medical, Guangzhou, China) was injected through the elbow vein with a double syringe, with a dose of 0.1 ml/kg body weight and a rate of 2-3 mL/s.

### 2.3 ^18^F-FDG PET/CT examination

Philips Gemini TF 64 PET/CT imaging equipment was used, and the imaging agent ^18^F-FDG was provided by Guangzhou Atomic High-tech Co., LTD. The radiochemical purity was >95%. Subjects fasted for more than 6 h, blood glucose was controlled below 8.0 mmol/L, and ^18^F-FDG was injected intravenously at 0.12 mCi/kg. Then, PET/CT examination was performed from the cranial top to the bilateral upper femur. PET/CT images were acquired under the following settings: 6-8 bed (acquisition time was 3 min/bed), FOV 600 mm, matrix 512×512, 2-mm slice thickness, 3D mode. A low dose non-contrast CT scan was acquired first and used for attenuation correction as well as an anatomical guide. CT was performed with 140 kVP, 200 mAs and 2mm slice thickness. The PET data were reconstructed using attenuation correction and an iterative reconstruction method. After image reconstruction, axial, coronal, sagittal and fusion images were obtained.

### 2.4 Image processing and data analysis

**Figure 1:**
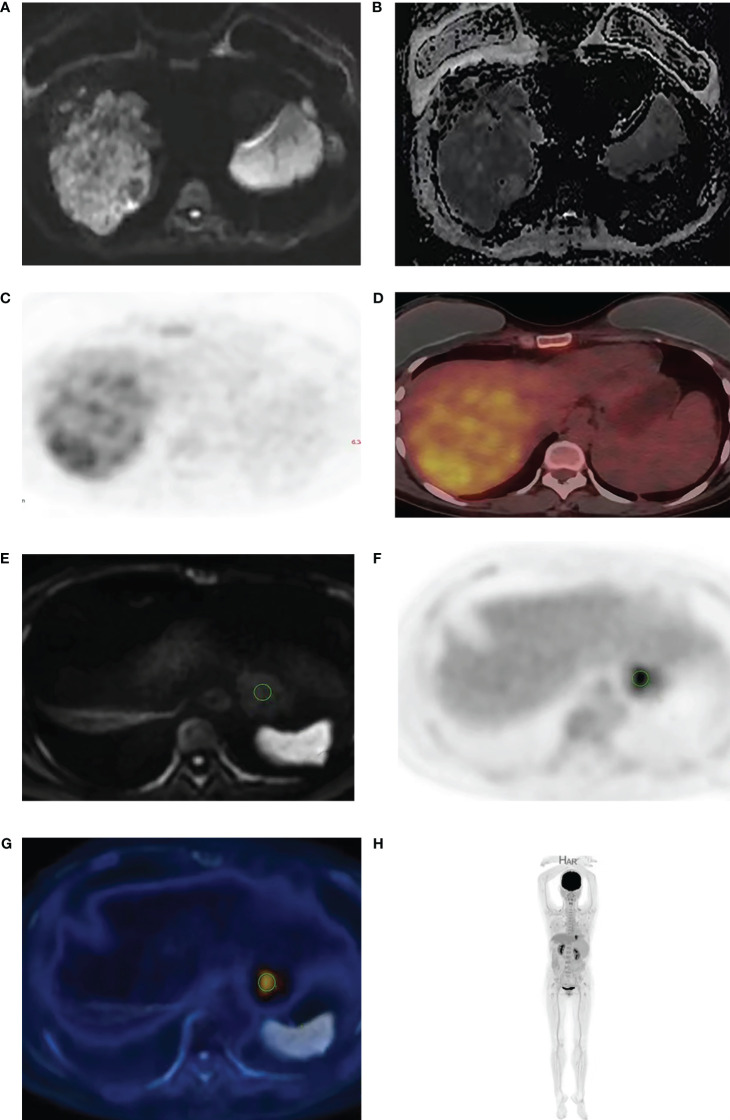
A 53-year-old man with HCC. The high b value (b=2000) diffusion weighted image **(A)** shows high signal intensity in HCC with corresponding low signal in the ADC maps **(B)**. ^18^F-FDG PET **(C)** and fused PET/DWI **(D)** images show strong 18F-FDG uptake. The delineation of ROI is achieved through the integration of different machines **(E–H)**.

Axial DWI images were imported into the ADW4.5 workstation by two imaging doctors with 4 years and 10 years of MRI and ^18^F-FDG PET/CT diagnostic experience, respectively. They analyzed the images separately and completed data measurements without knowing the pathological results. The layer with the largest lesion was selected, and the layer with the most solid components in the arterial phase images of the LAVA sequence was selected as the outline of interest. Attention was given to avoid the areas of necrosis, cystic degeneration and hemorrhage (T1WI, T2WI and LAVA were compared with each other), and an ROI of approximately 1.0 cm2 was placed on each of the three adjacent layers. The three average values were used for data analysis (**Figures 1A–D**). Fusion through different planes ensure that the positions outlined in the ^18^F-FDG PET-CT and DWI images were on the same plane, the same number and size of ROIs were placed in the same position (**Figures 1E–H**).

### 2.5 Determination of immune index expression

**Figure 2:**
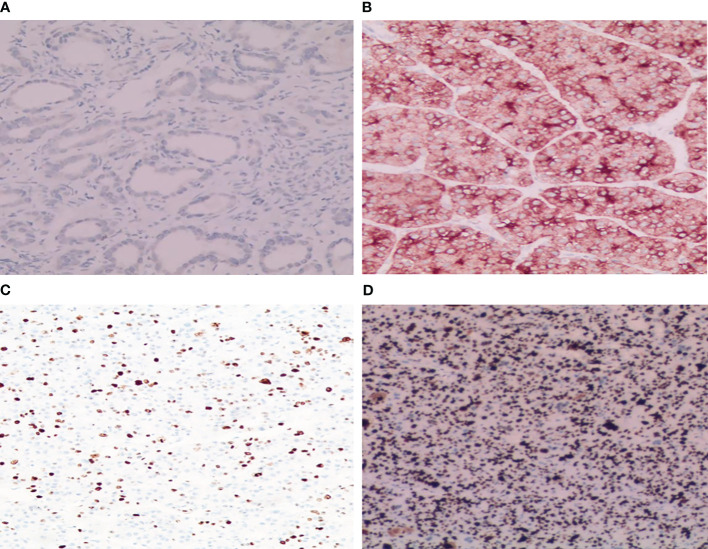
Immunohistochemical staining results of GPC-3 and Ki-67. **(A)** Tumor cytoplasm was weakly stained and GPC-3 negative (HE×200); **(B)** Tumor cytoplasm was strongly stained and GPC-3 positive (HE×200). **(C)** A few dark brown particles were found in the nucleus of the tumor with low expression of Ki-67 (8%+) (HE×100). **(D)** A large number of dark brown particles were found in the nucleus of the tumor with high expression of Ki-67 (90%+) (HE×100).

GPC-3 staining was performed using a Roche Ventanta BenchMark ULTRA Automatic immunohistochemistry analyzer. We adopted the comprehensive scoring system proposed by Takai (8), which consists of three factors, including positive cell rate, staining intensity and staining mode. Positive cell rates were classified according to the grades of 0 to 3 as follows: 0 (< 5%), 1 + (5-10%), 2 + (10-50%) and 3 + (> 50%). Staining scores of 2 and 3 were defined as positive staining, while 0 and 1 were defined as negative staining. All biopsies were reviewed by two independent pathologists in accordance with the World Health Organization (WHO) standard guidelines (9) (**Figures 2A, B**). Ki-67 expression was located in the tumor nucleus, and the positive criterion for its expression was that clear brownish-yellow particles appeared in the cytoplasm of tumor cells, and its staining degree was higher than that of the nonspecific staining background. The mean positive percentage of 2000 tumor cells was counted by three pathologists in the area with the strongest marker (hot spot). According to relevant standards (10–12), Ki-67 expression index >10% was considered the high-expression group, and Ki-67 expression index ≤10% was considered the low-expression group (**Figures 2C, D**).

### 2.6 Statistical analysis

SPSS 22.0, MedCalc (V20.0.3) and GraphPad Prism 9 statistical software were used for data analysis. The classified data were analyzed by the chi-square test and expressed as frequencies and percentages. The Shapiro-Wilk(S-W) test was used to test the normality of all measurement data. Those with a normal distribution are represented by *x* ± s, and those with a nonnormal distribution are represented by the median (upper and lower quartiles). Intraclass correlation coefficients (ICCs) were used to test the consistency of each parameter of the two doctors (ICC<0.40 indicated poor consistency, 0.40≤ICC<0.75 indicated medium consistency, and ICC≥0.75 indicated good consistency). The average of the two doctors’ measurements was analyzed. According to a normal distribution, the independent sample T test or Mann−Whitney U test was used to compare the difference in each parameter value between the Ki-67 high- and low-expression groups and the GPC3 group. Binary logistic regression was used to analyze the combined Apparent diffusion coefficient (ADC) and SUVmax values. Receiver operating characteristic (ROC) curves were used to evaluate the identification efficiency of parameters with significant differences for each group of lesions, and the area under the curve (AUC) was calculated. The boundary value, sensitivity and specificity were determined according to the most approximate index. The Delong test was used to compare the AUC values of the combined and single parameters. Pearson (in line with normal distribution) or Spearman (in line with abnormal distribution) correlation analysis was used to analyze the correlation between the parameter values with significant difference and Ki-67 expression index (correlation coefficient R was between -1 and 1, 0≤ ∣ R∣ <0.20 was no correlation or very weak correlation. For example, 0.20≤ ∣ R∣ <0.40 was weak, 0.40≤ ∣ R∣ <0.60 was medium, 0.60≤ ∣ R∣ <0.80 was strong, and 0.80≤ ∣ R∣ ≤1 was extremely strong). P<0.05 indicated that the difference was statistically significant.

## 3 Results

### 3.1 General clinicopathological data

A total of 94 HCC patients were included in this study (51 patients in the Ki-67 high-expression group and 43 patients in the Ki-67 low-expression group; 49 patients in the GPC-3-positive group and 45 patients in the GPC-3-negative group) as shown in **Table 1**.

**Table 1:** General clinicopathological information of the HCC patients.

Clinical features		Ki-67			GPC-3		
		High expression (n = 51)	Lower expression (n = 43)	*P value*	Positive group (n = 49)	Negative group (n=45)	*P value*
Gender, n (%)	male	45 (88.23)	40 (93.02)	> 0.05	44 (89.8)	41 (91.11)	> 0.05
	female	6 (11.77)	3 (6.98)		5 (10.2)	4 (8.89)	
Age (y)		57.65 ± 9.66	59.47 ± 8.71	0.344	57.45± 8.59	60.02± 9.75	0.177
Maximum diameter (cm)		47.25 ± 35.58	44.77 ± 27.71	0.710	45.24 ± 32.29	45.58± 27.37	0.957
ALT (U/L)		40.93± 40.97	42.12 ± 23.19	0.860	42.51± 24.26	40.42 ± 40.08	0.758
AST (U/L)		40.49 ± 23.77	44.39± 15.69	0.343	44.41 ± 15.49	40.49 ± 24.26	0.349
GGT (U/L)		111.35± 239.73	106.14 ± 108.04	0.889	107.06 ± 109.85	108.87± 234.59	0.961
TBIL (mu mol/L)		15.37 ± 7.04	15.68 ± 7.41	0.833	16.20± 7.77	12.80 ± 5.69	0.019
ALB(g/L)		39.37 ± 4.01	38.26± 8.06	0.416	36.14± 10.47	39.51± 3.87	0.045
AFP(ng/mL)		9993.30 ± 27154.39	997.53 ± 1554.59	0.032	13157.33 ± 37902.11	1274.95 ± 2689.53	0.039
Cirrhosis of liver n (%)	yes	36 (70.59)	28 (65.12)	> 0.05	33 (67.35)	29 (64.44)	> 0.05
	no	15 (29.41)	15 (34.88)		16 (32.65)	16 (35.56)	
Number of tumors n(%)	single	34 (66.67)	27 (62.79)	> 0.05	31 (63.27)	30 (66.67)	> 0.05
	multiple	17 (33.33)	16 (37.21)		18 (36.73)	15 (33.33)	

### 3.2 Consistency test of the measurement results of two doctors’ parameters

The two doctors analyzed the lesion ADC value and SUVmax of the Ki-67 and GPC-3 expression group. The ICC values were all greater than 0.80.

### 3.3 Comparison of parameters between the two groups

**Figure 3:**
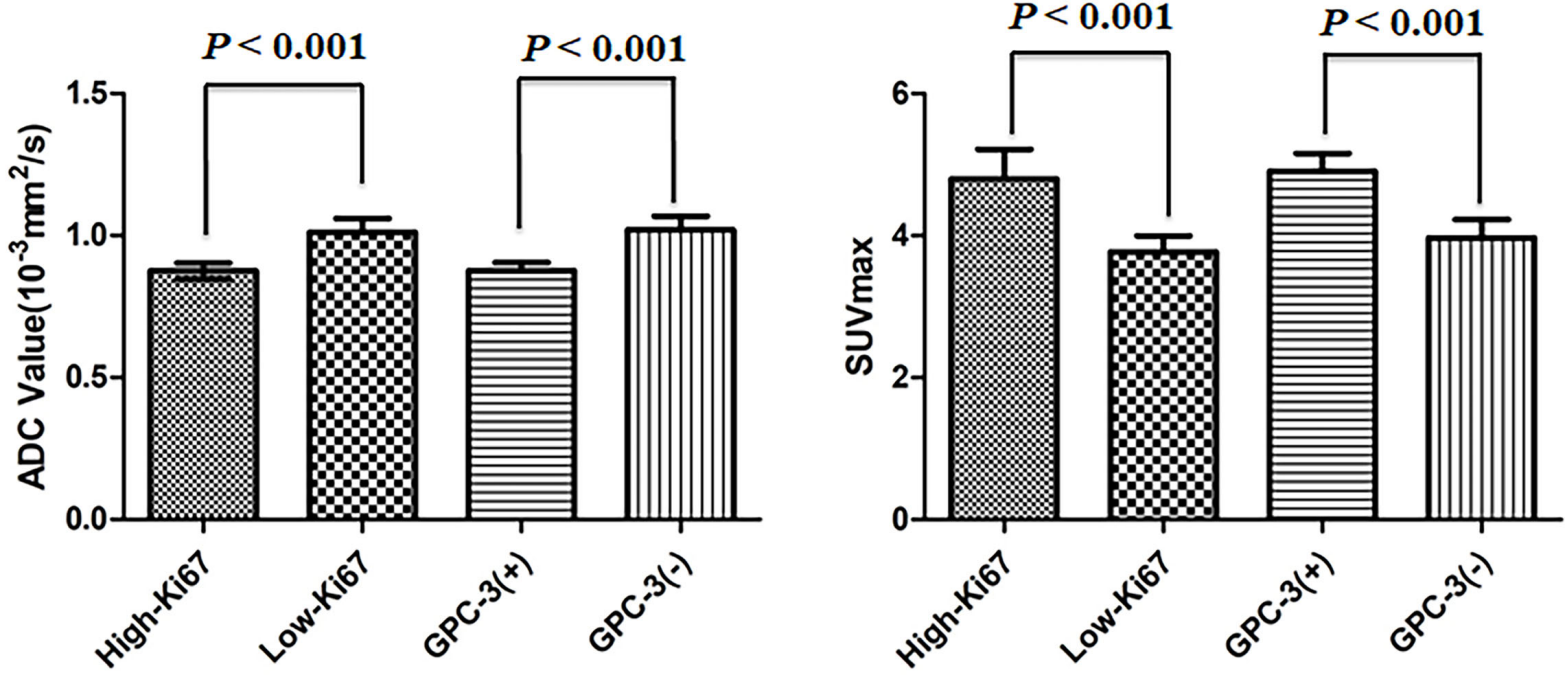
Error bars and 95% CI of high- and low-Ki-67-expression groups and positive- and negative-Glypican (GPC)-3 groups.

The ADC value of the Ki-67 high-expression group was lower than that of the low-expression group (*P*<0.05), and the SUVmax value of the Ki-67 high-expression group was higher than that of the low-expression group (*P*<0.05). The ADC value of the GPC-3-positive group was lower than that of the GPC-3-negative group (*P*<0.05), and the SUVmax value of the GPC-3-positive group was higher than that of the GPC-3-negative group (*P*<0.05) as shown in **Figure 3**; **Table 2**.

**Table 2:** Comparison of ADC (10^-3^ mm2/s) and SUVmax values between the Ki-67 and GPC-3 groups.

	Ki-67				GPC-3			
	High expression (n=51)	Low expression (n=43)	*T/Z value*	*P values*	Positive (n = 49)	Negative (n=45)	*T/Z value*	*P values*
ADC value	0.88 ± 0.10	0.98 (0.92, 1.16)	4.80	< 0.001	0.88 ± 0.10	0.96 (0.90, 1.14)	4.33	< 0.001
SUVmax value	4.5 (3.7, 5.8)	3.8 (3.2, 4.3)	3.61	< 0.001	4.9 ± 0.88	4.0 ± 0.87	5.21	< 0.001

### 3.4 Diagnostic efficiency evaluation of each parameter value

**Figure 4:**
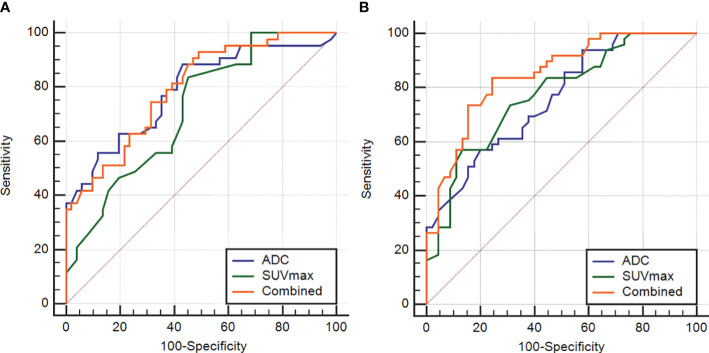
The ROC curves of the ADC and SUVmax values combined in the Ki-67 and GPC-3 groups **(A)**, **(B)**.

The AUC, threshold, sensitivity and specificity of the combined diagnosis were identified by each parameter in the Ki-67 and GPC-3 groups as shown in **Table 3**. ROC curves are shown in **Figure 4**. The comparison of the parameter values between Ki-67 and GPC-3 and the AUC of the combined model is shown in **Table 4**.

**Table 3:** AUC, threshold, sensitivity (%) and specificity (%) of parameters of the Ki-67 and GPC-3 groups.

	Ki-67				GPC-3			
	AUC	threshold	sensitivity	Specificity	AUC	threshold	sensitivity	Specificity
ADC value	0.788	0.883	88.4%	56.9%	0.759	0.89	80%	57.1%
SUVmax value	0.716	4.45	54.9%	83.7%	0.773	4.65	57.1%	86.7%
ADC+ SUVmax value	0.796	0.434	86.3%	51.2%	0.847	0.51	75.6%	83.7%

**Table 4:** Comparison of parameter values between Ki-67 and GPC-3 and the AUC of the combined model.

	Ki-67	GPC-3
	*P* values	*P* values
ADC VS SUVmax value	0.1963	0.8307
Combine model VS SUVmax value	0.063	0.0324
Combined model VS ADC values	0.679	0.0437

### 3.5 Correlation analysis of SUVmax, ADC and Ki-67 values

**Figure 5:**
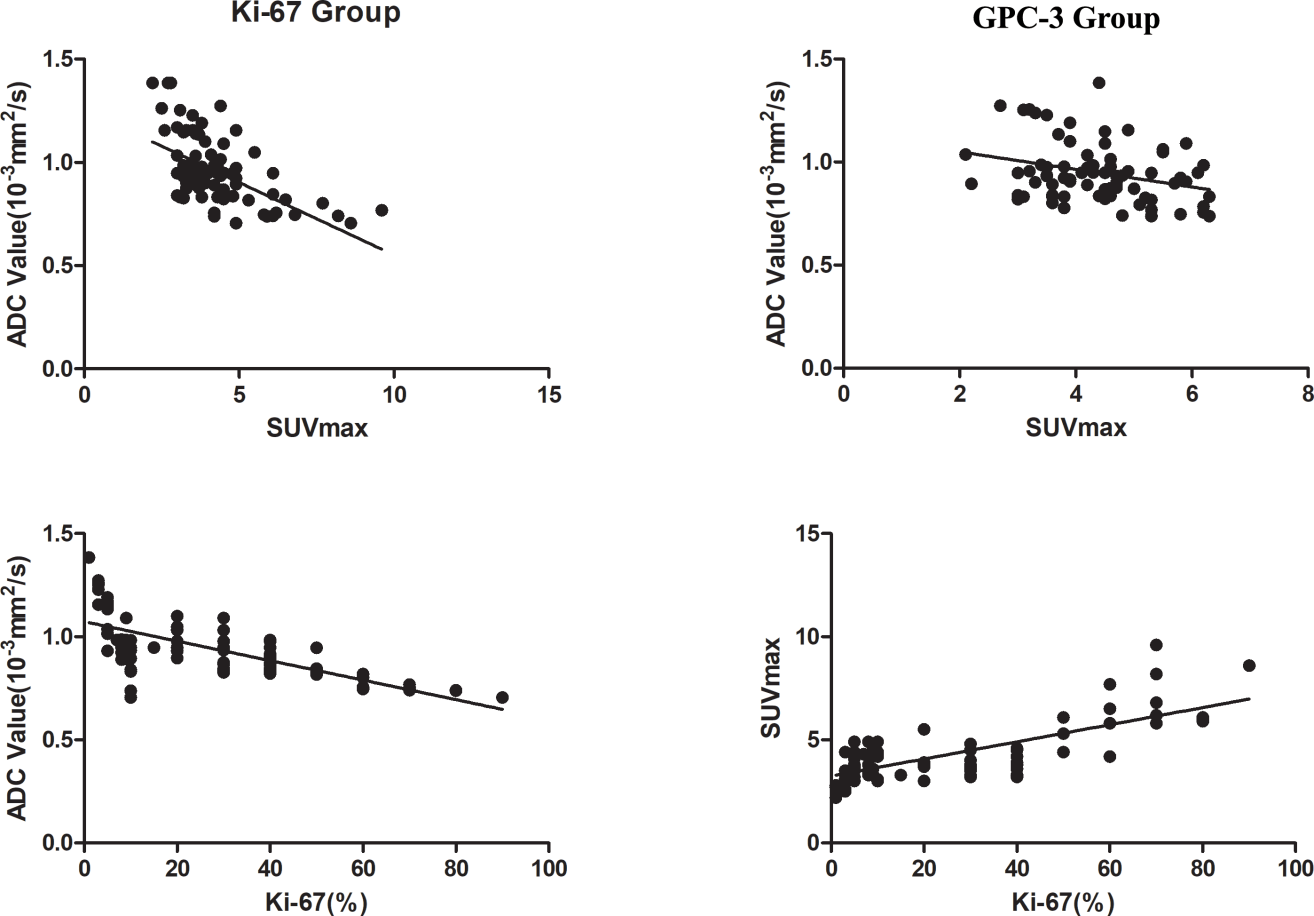
Correlation analysis chart of ADC with SUVmax values in the two groups and Ki-67 with ADC and SUVmax values.

There was a strong negative correlation between the SUVmax value and ADC value in the Ki-67 group (R=-0.578, *P*<0.001and a weak negative correlation between the SUVmax value and ADC value in the GPC-3 group (R=-0.279, *P*=0.006). The SUVmax value was strongly positively correlated with the Ki-67 expression index (R=0.733, *P*<0.001), while the ADC value was strongly negatively correlated with the Ki-67 expression index (R=-0.687, *P*<0.001) as shown in **Figure 5**.

## 4 Discussion

Our results showed that both the ADC and SUVmax values can quantitatively evaluate the expression of Ki-67 and GPC-3 in HCC patients, and the higher the expression of Ki-67, the higher the SUVmax value and the lower the ADC value. The combination of DWI and PET-CT can improve the diagnostic efficiency and benefit patients in the GPC-3 group. In addition, we found that the SUVmax and ADC values had a strong negative correlation in the Ki-67 group and a weak negative correlation in the GPC-3 group.

Huang (13) found that the ADC value of liver cancer was negatively correlated with the expression of Ki-67 (R=-0.371). The results in this paper were roughly similar to previous results, but our correlation was stronger (R =-0.687), possibly due to our larger sample size, so the correlation was higher. Gu (14) and Chong (15) found that a radiomics nomogram model based on MRI can achieve preoperative prediction of GPC-3 expression in HCC. Zhao (16) found that the 75% ADC value could help distinguish GPC-3-positive and GPC-3-negative expression states based on a texture histogram of DWI. Chen (17) evaluated the GPC-3 expression state by Iterative decomposition of water and fat with echo asymmetry and least-squares estimation-iron sequence (IDEAL-IQ). The results showed that R2* could be used to predict GPC-3 expression in HCC. We also found that the prediction of the GPC-3 expression state could be achieved by using the ADC value, which was consistent with previous results indicating that MRI-related technology could be used to measure GPC-3 expression noninvasively before surgery.

Kitamura (18) showed that the SUVmax value of HCC increased with increasing Ki-67 expression; unfortunately, they did not carry out correlation analysis. The research results of this paper also further confirm that the SUVmax value can be used to evaluate the expression of Ki-67, and the correlation between them was further analyzed. Our results are consistent with many previous studies (19–21). This finding indicates that the SUVmax value can be used as a quantitative method for evaluating the expression of GPC-3 in HCC.

The results of two meta-analyses (22, 23) show that ^18^F-FDG PET/CT and MRI are comparable in evaluating the diagnostic efficacy of lymph node metastasis in breast cancer and in evaluating the therapeutic efficacy of pancreatic cancer; unfortunately, the combination and comparison of the two technologies have not been conducted. In this article, the SUVmax and ADC values could be used to achieve noninvasive preoperative prediction of the Ki-67 and GPC-3 expression status and determine which imaging method was better. There is no unified conclusion at present, and our results indicate that the diagnostic efficacy of the two imaging techniques is the same. However, our findings indicate that the combination of the two imaging techniques is better than a single technique in the GPC-3 group. Therefore, we have reason to believe that preoperative evaluation of the immunohistochemical expression status of HCC by the combination of the two techniques can benefit patients. Qiao (24) used ^18^F-FDG PET/CT and DSC-PWI to assess tumor recurrence and radiation damage in patients with high-grade gliomas. Their results showed that the combination of the two techniques improved diagnostic accuracy. ^18^F-FDG PET/CT combined with MRI also showed that the combination of the two techniques was superior to the single technique in assessing breast cancer staging (25). The results of the multisystem study and our study show that ^18^F-FDG PET/CT combined with MRI can benefit the treatment and prognosis of patients.

Byun (26) and So-Yeon Lee (27) found a negative correlation between SUVmax and ADC values in the evaluation of musculoskeletal tumors. Hong (28) used ^18^F-FDG PET/CT and DWI to evaluate the prognosis of HCC, and their results showed a negative correlation between the average SUVmax value and ADC value of tumors (R = -0.402). We also found that there was a correlation between SUVmax and ADC values, and the correlation was higher for Ki-67 expression (R=-0.578). This may be because Ki-67 mainly reflects the proliferation activity of cells, thus acting as a nuclear antigen related to proliferating cells to judge the degree of malignancy of tumors. The SUVmax value increases with increasing glucose metabolism and malignancy of tumors, and the ADC value has been widely used in the differentiation of benign and malignant tumors. The Ki-67, SUVmax and ADC values can indicate the degree of malignancy of tumors, so they are highly correlated. GPC-3, as a glycoprotein, is mainly present in the serum of HCC patients and plays a role mainly through the regulation of the Wnt signaling pathway, while the expression product of the Wnt gene can promote the growth of various tumor tissues (29). GPC-3 is mostly used as an early diagnostic marker of HCC, and there is no clear evidence of the relationship between GPC-3 and the degree of malignancy of tumors, while SUVmax and ADC values are used as semiquantitative indicators of the degree of malignancy of tumors, so this may be due to this reason. As a result, the correlation between SUVmax and ADC values in the GPC-3 group was low (R=-0.279).

The results of this study found that Total bilirubin (TBIL) and Albumin (ALB) can identify positive- and negative GPC-3 group. As indicators of liver function, TBIL and ALB were mainly used to assess whether there was liver disease or damage, with many influencing factors and little relationship with the occurrence and development of HCC. The results of multiple small sample studies found that TBIL and ALB in different GPC-3 groups have not been uniformly concluded, and further confirmation requires multi-center and large sample studies (14, 30, 31). AFP was significantly different in Ki-67 and GPC-3 groups. AFP, as a serological tumor marker for HCC, has been widely used in clinical work. AFP has also been proved to promote tumor cell proliferation and inhibit cell apoptosis, and has an important relationship with the occurrence and development of HCC. Wang (31) et al. also found that AFP was statistically different in GPC-3 and Ki-67 groups, which was the same as our results, suggesting that AFP could be used as a non-invasive method to identify positive GPC-3 and high expression of Ki-67.

Our study has several limitations. 1: Our study is a single-center retrospective study, which requires follow-up verification with a large sample size from multiple centers. 2: The delineation of multiple ROIs in MRI and ^18^F-FDG PET/CT may not be able to be achieved completely at one level, causing certain deviations. 3: Different histological types and grades of HCC were found in ADC and SUVmax. There may be differences between the values, but due to the limited sample size, this study did not conduct a specific grouping study on histological types and grades. 4: ROI area placement may not be able to completely correspond with immunohistochemical sampling.

DWI and ^18^F-FDG PET/CT can be used to evaluate the expression of Ki-67 and GPC-3 in HCC, and there is a certain correlation between the ADC value and SUVmax. The two technologies can complement each other and benefit patients in the GPC-3 group. However, the combined model did not benefit the patients in the Ki-67 group.

## Data availability statement

The raw data supporting the conclusions of this article will be made available by the authors, without undue reservation.

## Ethics statement

Written informed consent was not obtained from the individual(s) for the publication of any potentially identifiable images or data included in this article.

## Author contributions

XW contributed to the study’s conception and design. Material preparation, data collection, and analysis were performed by LL. The first draft of the manuscript was written by XW and was revised by MC. All authors contributed to the article and approved the submitted version.
